# Dwell-photo nodes in classical garden tourism: a multimodal spatial analysis of Yipu

**DOI:** 10.3389/fpsyg.2026.1772537

**Published:** 2026-06-09

**Authors:** Fang Zhang, Zhide Wu, Xi Zhou

**Affiliations:** School of Architecture and Urban Planning, Suzhou University of Science and Technology, Suzhou, China

**Keywords:** classical garden, Dwell-Photo node, experience, pedestrian simulation, tourism, visual perception

## Abstract

Sensory experiences sharing in the social media era has expanded tourism’s social influence, and visual experiences are critical for connecting visitors with specific locations. With the widespread adoption of information technology and the internet, visitors’ intentions have moved from traditional sightseeing to a hybrid experiential loop of “recording, sharing and re-disseminating.” In this process, spatial and perceptual experiences are transformed into communicable social information via Dwell-Photo (DP) routine, providing crucial feedback for the spatial design and renewal of heritage spaces. Jiangnan classical gardens, with their complicated spatial systems and rich cultural value, offer compelling cases for examining the mechanics that drive DP behavior. This study employed the classical garden Yipu, also known as the Garden of Cultivation, for representative samples of DP nodes to create a research framework based on multimodal data that integrates pedestrian simulation and visual perception methodologies with traditional investigative approaches. By developing an associative model between spatial experience perception and DP behavior, this study delves deeply into the patterns of tourists’ visual perception and touring behaviors in classical gardens, accurately extracting the cultural and aesthetic essence of garden environments. This presents a viable technical avenue for classical gardens to transition from “sightseeing check-in” to immersive cultural experiences, and the proposed methodological framework is intended to provide critical input for the spatial design and maintenance of heritage spaces. By connecting spatial perception data with tourism planning and management plans, avoiding resource waste due to subjective judgments, and offering decision-making references for tourism planning and management, its expanded application can incorporate humanistic elements into urban street view aesthetic design.

## 1 Introduction

The Jiangnan classical gardens are regarded as an immersive creative paradigm that, especially in the modern media era, reflect a distinctly eastern aesthetic in both formal aesthetics and perceptual experience (Lu, 2016). As a living example of Chinese architectural culture, Jiangnan classical gardens are distinguished by their use of spatial form as a means of immersive interaction. In the media age, their underlying Chinese spatial aesthetics have been revitalized through social platforms, where rapid documentation and sharing create a dynamic loop of “Experience, Dwell-Photo check-in, and Dissemination.” In addition to on-site DP behavior (Dwell-Photo check-in activity), visitors’ sensory immersion and spatial perception within the gardens manifest through multimodal digital expressions including texts, images, and short videos, which consistently increase the gardens’ visibility and cultural resonance in online environments.

As a UNESCO World Heritage Site and a well-liked tourist site, Suzhou’s classical gardens have faced both significant challenges and opportunity for widespread dissemination in the media era. On the one hand, as a tourist destination, Suzhou classical gardens have ushered in unprecedented opportunities for dissemination and experience upgrading in the media era, particularly in the dual role of tourists’ spatial experience and media communication, truly realizing the two-way pursuit of “Eastern aesthetics” and “Network flow.” In this process, the experience transformation produced by visual perception has supported the value upgrading of destination culture: classical gardens are no longer just “tourist attractions” for visitors to enjoy, but rather “cultural carriers” transporting the beauty of Eastern living. When this type of dissemination occurs on a large scale, a large number of tourists, through visual capture and deepening perception, promote the destination culture from a short-term tourist memory to a shared spiritual resonance(Adriani et al., 2021; Ai et al., 2022), achieving the value transition from “landscape consumption” to “cultural identity.” On the other hand, in the current era of digital communication technology reshaping the cultural and tourism ecology, the phenomenon of “sightseeing check-in” in classical gardens has become an important entry point for observing the dissemination of traditional culture and the enhancement of visitors experience, and its value potential has yet to be fully explored. It is critical to conduct scientific research on the phenomena of classical garden check-in and DP behavior, which is not only the key to improving the quality of visitor experience, but also an important pathway for promoting the creative transformation and inventive development of heritage space.

In response to this phenomena, previous research has primarily portrayed check-in behavior as surface consumerism, however this study focuses on the intrinsic linkage and transformation process between “Dwell” and “Photo” behaviors. In-depth analysis of tourists’ visual perception patterns and travel behavior characteristics in classical gardens, precise extraction of the cultural aesthetic core of garden space, can provide a practical technical path for classical gardens to achieve a value upgrade experience from “sightseeing check-in” to “cultural immersion.” Through Pedestrian Simulation and Visual Perception Experiment, it examines the spatial characteristics of visitors’ “Dwell” nodes during sightseeing, as well as their attention allocation throughout the check-in “Photo” process, and investigates the triggering factors from “Dwell” to check-in “Photo.” The research aims to reveal the underlying experiential needs that drive DP behavior, giving forth a transformative path from “media-fueled tourism draw” to “cultural values transmission” in landscape design, and offering critical feedback for the spatial design and renewal of heritage spaces.

## 2 Research development of tourist experience

Environmental psychology, an interdisciplinary area focusing on the interaction of humans and the environment, offers broad theoretical support and practical direction for the study of tourist experience in architecture and urban planning. Its core theories, such as environmental cognition theory, reveal the underlying logic of tourist perception and understanding of architecture and urban space, assisting researchers in analyzing how tourists form impressions of their destinations through spatial cognition and providing a theoretical foundation for optimizing spatial layout to improve visitors memory. On a practical level, behavioral psychology’s study of tourist engagement psychology has accelerated the transition from “space-centered” to “visitor-centered” methods in architecture and urban planning, offering quantitative benchmarks for spatial development. With the expansion of multidisciplinary research, new approaches and technology tools have arisen as scientific instruments for visitor experience research.

With the advancement of technology, spatial environmental technologies, which offer integrated data processing, modeling, and visualization capabilities, have emerged as essential instruments for studying spatial behavior. These technologies greatly improve our comprehension of the principles behind interactions between human behavior and geographical surroundings through spatiotemporal data modeling and dynamic simulation. In recent years, GIS (Geographic Information Systems) and spatial syntax have made it possible to quantitatively decode spatial patterns in architectural and urban studies, converting qualitative descriptions of gardens into verifiable spatial data and offering important insights into spatial order and human-centered design (Bafna, 2003; Conroy-Dalton, 2005; Zhang and Zhou, 2023). The use of axial maps, visibility graphs, and multi-temporal spatial analyses in revealing relationships between garden spatial structure and visitor behavior has made the integration of space syntax and GIS a new frontier in garden and heritage research (Huang et al., 2025). However, current applications tend to be primarily concerned with spatial structure and circulation, focusing on integration, accessibility, and visibility metrics while paying little attention to emotional involvement and perceptual reactions within particular spatial encounters (Yang and Zhang, 2018). With the expansion of modal data, technological integration has become a primary focus, and multimodal semantic analysis will combine data such as photographs and videos to more thoroughly depict tourists’ spatial experiences. Behavioral simulation technology can help scenic sites dynamically change spatial management techniques, while eye tracking technology gives a quantitative tool for studying tourists’ visual perception patterns of space (Xu et al., 2024). Deepening interdisciplinary research can aid in the construction of a theoretical model of spatial features that interact with visitor experience perception, offering more accurate support for the optimum design and sustainable development of tourism spaces.

Behavioral simulation is a dynamic extension of urban space-use analysis that arose from research on public safety and transportation in the late 20th century. It simulates individual movement and interaction in complex contexts using pedestrian dynamics models and ABM (Agent-Based Modeling) (Helbing et al., 2000; Zhang et al., 2022), as well as software platforms including MassMotion, Legion, and VISSIM to provide real-time three-dimensional prediction (Still, 2014). MassMotion is especially useful for BIM/CAD interoperability and three-dimensional visualization, whereas Legion is more suited for analyzing high-density flows and signal-control scenarios. In contrast to the static structural analysis provided by space syntax, behavioral simulation produces time-series data on pedestrian density, route choice, and dwell duration, demonstrating the dynamic effects of spatial settings on behavior. Out-puts that involve trip time, density-speed relationships, and queue dynamics may be cross-validated using these methods to improve the robustness of simulation results (Batty, 2001). In recent years, simulation-based methods have been used extensively to assess circulation organization, node congestion, and viewing rhythm in historic districts and tourist destinations (Liu et al., 2024). The combination of behavioral modeling and spatial analysis, specifically adapted to the features of Jiangnan classical gardens, may provide important in-sights into the research of spatial design and visitor behavior mechanisms (Chen et al., 2024; Sun and Yan, 2023).

As environmental perception research in architectural and landscape disciplines has progressed, visual analysis has evolved as critical techniques to comprehending spatial experiences (Fisher, 1991). Visual analysis and geo-visual studies in architecture have progressed from two-dimensional visibility calculations to three-dimensional dynamic models that integrate GIS, virtual reality, and visual perception experiments, elucidating the experiential logic of spatial accessibility and viewpoint configuration (Schafer et al., 1977; Turner et al., 2001). The significance of multisensory perception in urban environments has increased as urban development has progressed, and design optimization now frequently uses simulation-driven assessment techniques (Aletta et al., 2016). Eye tracking technology employs optical imaging and computer vision to precisely record data including fixation point, fixation length, scanning path, and pupil changes. It can graphically display the distribution of tourists’ visual attention while also indirectly reflecting their cognitive load and emotional state (Jeon et al., 2011). This technology can quantitatively analyze tourists’ visual perception patterns of garden spaces, identify the most appealing landscape nodes or easily overlooked areas, reveal the guiding role of spatial layout and streamline design on the line of sight, and provide data support for optimizing the garden space experience (Goldberg and Kotval, 1999; Li, 2018). However, there is still a lack of comprehensive research on the mechanisms that link visitors’ gaze patterns to architectural approaches such as Framed-Views, Horizontal-Vertical Composition, or Light-Shadow Rhythm in Jiangnan classical gardens. There is currently a scarcity of extensive research and empirical validation on how perceptual experience translates into media-driven spatial engagement, particularly in today’s “Experience, Dwell-Photo check-in, and Dissemination” interaction.

## 3 Experience—perception of Jiangnan classical gardens

As the pinnacle of Eastern architectural and landscape traditions, the Jiangnan classical gardens have long been regarded as essential subjects of study in the fields of architecture and landscape (Zhou, 1999). Scholarly focus has gradually moved from historical and artistic analyses of garden-making principles to empirical research on visitor experience and behavior patterns as human-environment studies and environmental psychology have grown in popularity (Luo and Chen, 1999). These studies have been utilized to examine visitors’ movement trajectories, living patterns, and visual cognition inside garden spaces. They are often grounded in environment-behavior theory and landscape perception (Yu et al., 2001). From aesthetic representation of space to spatial construction of behavior, this step signifies a paradigm change in garden research.

In the digital age, spatial experience extends beyond instantaneous on-site perception. “Experience, check-in photo, and share” has improved the wandering experience in classical gardens as a consequence of picture capture, social media sharing, and internet distribution. In the context of social media communication, DP nodes are characterized as major spatial nodes within gardens or streetscapes where visitors prefer to slow down, pause, look around, and take photographs, and that demonstrate both obvious on-site clustering and high potential for online sharing and dissemination. Individual perceptual actions become intricately linked to the social and communicative aspects of space during the sharing process, creating new kinds of spatial meaning and collective memory. “A person’s immediate or sustained, subjective, and personalized psychological response to activities, environments, or events outside of their daily environment” is the definition of a tourist experience (Packer and Ballantyne, 2016). This concept, which goes beyond straightforward “satisfaction” metrics and suggests more complex perceptual structures, highlights its emotional, situational, and individual variations.

The DP behavior (Dwell-Photo check-in activity) in gardens has transcended traditional tourism category in the media era and evolved into a composite behavior that blends social communication, cultural expression, and spatial experience. From a behavioral perspective, the term “Dwell” refers to visitors’ active presence in garden areas for recreational purposes, cultural resonance, or landscape appeal. The duration, frequency, and location selection are an immersive “on-site experience” that reflects the depth of individual perception of garden aesthetics and historical connotations (Gehl, 2011). However, “Photo” has more social display attributes, with “arrival shooting sharing” as the core process, completing “self-identity construction” and “circle identification” through social media, effectively turning garden space into social information in the digital age. In the process of spatial experience and communication, a space’s attractiveness is based on both its physical characteristics and its capacity to inspire people to remain and actively spread (Gehl, 2013). Accordingly, the study of DP nodes and related perceptual production mechanisms can provide a critical lens through which to view the evolution of the spatial experience in this changing setting. Dwelling, known as short-term staying behavior, is often described in landscape psychology and environment-behavior research as an individual’s temporary pause in a specific spatial space, driven by both perceptual attractiveness and psychological interest (Whyte, 1980). Dwelling and taking photos are scenarios involving DP behavior that often make up the on-site component of check-in behavior in the media dissemination process. It is regarded as one of the most direct and visible forms of human-environment interaction, driven by features such as visual focal points, spatial enclosure, path curvature, and environmental comfort (Kaplan and Kaplan, 1989). DP behavior thus reflects people’s cognitive processing and emotional engagement in addition to the space’s potential for physical attractiveness (Montello, 2007; Nasar, 1994). DP activity is considered a coupling node between perception and action in the realm of landscape perception, where visual experiences are transformed into socially expressive behaviors like staring, taking pictures, conversing, or relaxing (Carmona, 2021; Tuan, 1977).

In brief, DP nodes are spots where individuals frequently pause and cluster in response to social interaction cues, landscape views, or physical characteristics. Garden surroundings can be evaluated for perceptual appeal and behavioral stimulation based on the quantity and length of these dwelling episodes. Based on the characteristics of landscape architecture of Jiangnan Classical Gardens and tourist feedback on social media platforms, the following hypotheses can be proposed.

① Framed-view design induces DP behavior

In Jiangnan classical gardens, interface elements including circular moon gates, latticed windows, and pavilions provide framed vistas by deliberately limiting sightlines and modulating spatial openness and enclosure. Focused visual fields appear as a result of the framed-view design, encouraging users to stop, linger, and carry out photographic acts at certain nodes.

② Horizontal-vertical composition modulates DP behavior

Within the garden, the meandering garden axis, combined with the intricate interplay of horizontal and vertical spatial relationships, generating landscape features composed in either horizontal or vertical layout. This compositional interplay modulates gait and pacing, leading visitors to slow or pause at points of turning, screening, or visual interruption.

③ Light-shadow rhythm stimulates DP behavior

The alternation of shade and illumination along covered corridors produces dynamic luminance variations that enhance visual stratification and perceived spatial depth. These rhythmic transitions intensify perceptual contrast, prompting longer fixation duration and stronger exploratory tendencies in areas where light-shadow conditions fluctuate frequently. Locations exhibiting dramatic light and shadow variations tend to become popular DP nodes.

## 4 Research design and methodology

**FIGURE 1:**
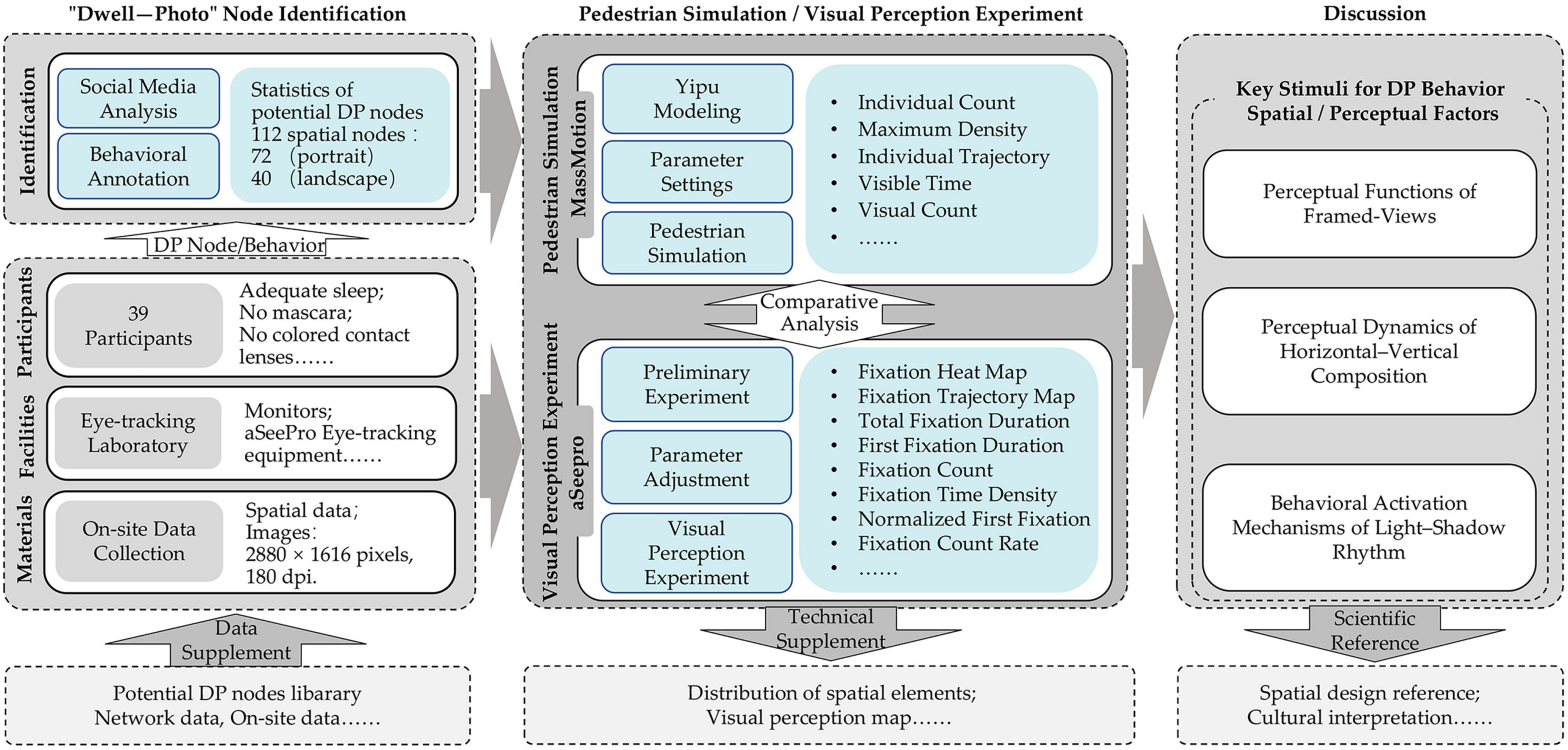
Framework of DP node analysis.

Yipu was chosen as a sample garden for empirical research in order to better comprehend and carry out in-depth research on the DP behavior of Jiangnan gardens. Using social media analysis and on-site behavior annotation, DP nodes were initially identified and verified, producing a set of sample nodes for measurement. Key behavioral characteristics such as dwell duration, activity type, and movement trajectory were well documented. Following that, simulation modeling and eye-tracking experiments were performed to characterize the spatial and perceptual properties of the DP nodes. Finally, by comparing the simulation and eye-tracking results, the hypothesis was supported, and the interaction mechanisms between spatial attraction, attention distribution, and emotional reaction in classical garden scenarios were studied (Figure 1).

① Identification: This process adopts social media analysis and behavior annotation. Using social media analysis, this study identifies spatial nodes with viral online popularity by scraping and analyzing high-frequency “photo check-in” content on social networking platforms, giving data support for future research sample (DP nodes) selection. To ensure the scientificity and accuracy of the research, on-site behavior annotation is introduced to accurately label the behavioral attributes, environmental characteristics, etc. of potential DP points, providing a calibration basis for online data, correcting errors such as data fluctuations, and supplementing DP points that are not covered online, making the recognition results more in line with the real scene and providing reliable support for subsequent research.

② Pedestrian simulation : Using behavioral trajectory algorithms of MassMotion, it simulates visitor movement patterns and dwell preferences in gardens, discovering highly appealing “Dwell” nodes in terms of spatial physical form and behavioral interaction.

③ Visual perception experiment: Using the DP nodes selected by the preceding two methods, the impact mechanism of spatial attraction elements (Framed-Views, Horizontal-Vertical Composition, and Light-Shadow Rhythm) on attention is quantitatively analyzed by recording data such as gaze duration, visual focus distribution, and so on.

Among the three stage of the aforementioned research framework, three main methods are used in bidirectional verification and progressive supplementation: the cross results of social media analysis and pedestrian simulation can identify DP nodes and mutually confirm the popularity and spatial attractiveness of sample nodes; visual perception experiment reveal tourists’ micro perception patterns toward space, providing empirical evidence for optimizing tourism spaces and heritage tourism.

### 4.1 Study area

Jiangnan classical gardens are widely considered as the pinnacle of Chinese classical garden design, while Suzhou classical gardens, which are on the UNESCO World Heritage List, are regularly cited as outstanding patterns. These attractions have long been popular among visitors. According to the SMBC (Suzhou Municipal Bureau of Culture, Radio, Television and Tourism), Suzhou classical gardens recorded over 190 million visitor arrivals in 2024 (Suzhou Municipal Bureau of Culture Radio Television and Tourism, 2025), with 812,700 on the May Day holiday in 2025 (SuBaoNet, 2025). Suzhou classical gardens provide an appropriate empirical template for examining DP behavior due to its continued public significance and visitor attractiveness. However, it should be high-lighted that sample selection must strike a balance between experimental controllability and scientific rigor, ensuring that the sample size is appropriate and the samples are representative, so assuring the dependability of the results. Large scale gardens, such as the Humble Administrator’s Garden and the Lingering Garden, are more representative of layered spatial sequences and route design, yet their large size and dispersed visitation cause crowd magnitudes highly sensitive to holiday effects, resulting in overflow bias and reducing the accuracy of behavioral data capture. Yipu, also known as the *Garden of Cultivation*, is a small-scale garden that makes it easier to observe and track behavioral trends due to its compact design and steady visitor flow. The garden is well known for its distinguishing features, effective design, and a broad variety of cultivation methods.

**FIGURE 2:**
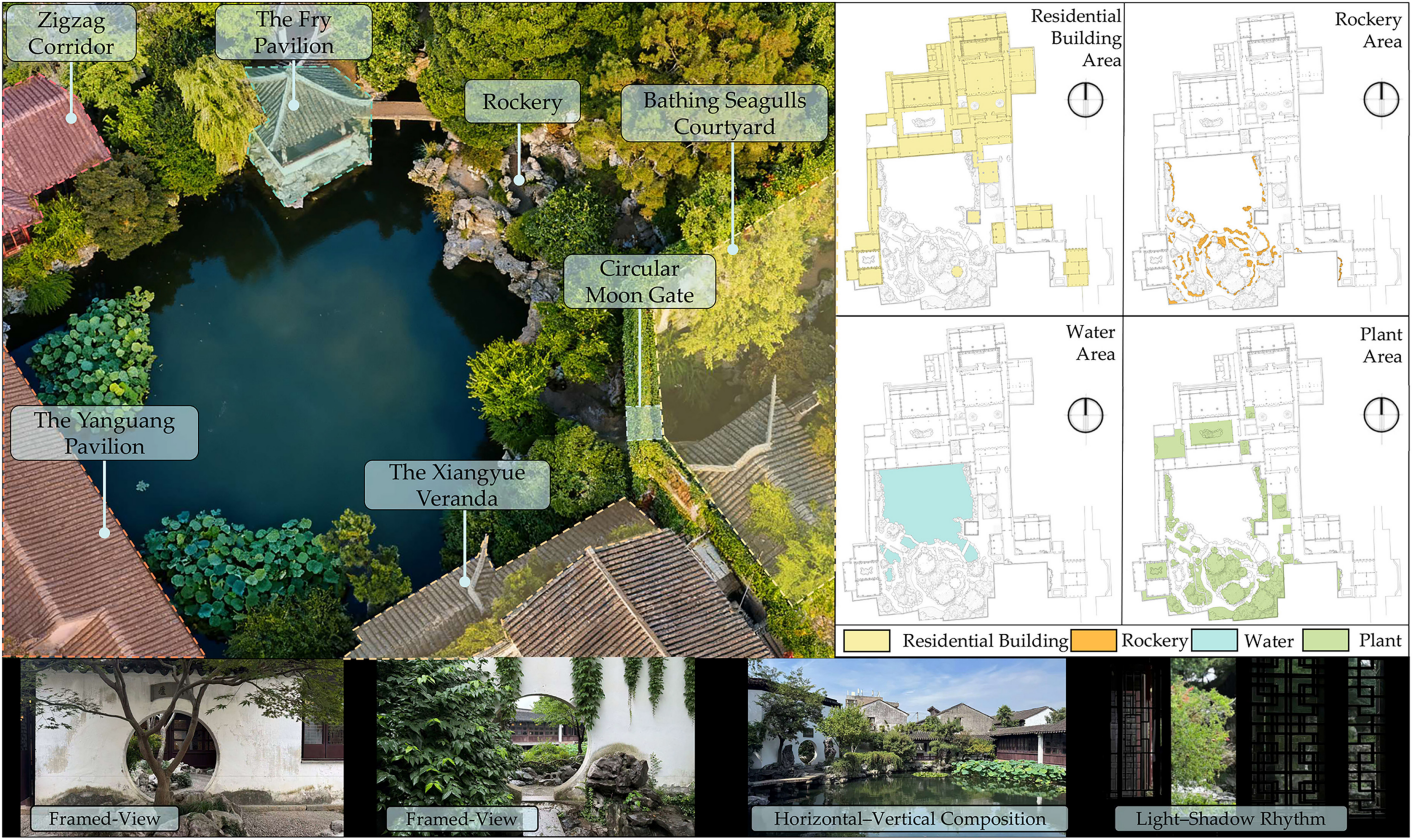
Spatial components and key nodes of Yipu.

The plot has around 3,300 m^2^, with 1,300 m^2^ dedicated to garden space. It expresses the unique spatial layering of Suzhou classical garden spaces and is a representation of the *Urban-site Garden* type described in *The Craft of Gardens* (Hardie, 1989). According to the *Yipu Gazetteer*, Wen Zhenmeng (son of Wen Zhengming) built the garden in 1,541, and literati cultivated and maintained it from the late Ming to the early Qing dynasty (Lin, 2013; Suzhou Municipal Administration of Gardens Greening, 2016; Wikipedia contributors, 2025). It follows the “residence on north with garden on south” layout in urban residential plots. The garden has retained the traditional characteristics of Jiangnan gardens since restoration and public reopening in 1984. In the age of networked dissemination and image-sharing platforms, Yipu has grown in popularity as an unofficial photography location, becoming a hub where visitors gather, dwell and take photos (Figure 2).

- Representativeness of visitor behavior: The compact layout of Yipu enables efficient, high-density recording of visitors’ spontaneous photography behavior and gaze patterns via eye-tracking, resulting in robust and representative data for research.
- Diversity of spatial samples: The master plan for the site is based on a central water spine that acts as the fundamental spatial axis, with rock formations, architectural elements, and seasonal flora ingeniously combined to create a highly layered environment with meandering pathways. This planned compositional design not only enhances the spatial experience, but also ensures a diverse range of visitor behaviors, notably DP interactions.
- Stability and richness of social media data: Over 10,300 Yipu-related image-text posts and comments were recorded on the RED (Rednote or Xiaohongshu) platform between 2024 and 2025, with 7,597 original posts in 2024 alone (RED, 2025). The platform’s strong diffusion potential for DP material is demonstrated by this steady and significant user involvement, which supports the representativeness of the behavioral data gathered.

### 4.2 Identification

#### 4.2.1 Identification of DP nodes

To provide a credible baseline and dataset for subsequent simulation experiments and eye-tracking investigations, the two-stage screening approach combines large-sample online data with fine-grained offline observation. A spatial model of Yipu is constructed, the distribution of potential DP nodes is recorded, and the high-frequency nodes of visitor photography and residency may be identified via social media data. On-site behavioral annotation, which documents visitors’ dwell periods, framing orientations (camera/view direction), and movement trajectories over multiple time frames, is then used to validate and adjust the results.

- Social media analysis: Within the contemporary digital media context, social media data are widely employed as a key basis for behavior investigation. By mining image-text posts and associated tag and hashtag metadata on social platforms, the spatiotemporal distribution and visual preferences of visitor can be characterized objectively, thereby providing external validation and input data for subsequent behavioral annotation and simulation experiments.
- Behavioral annotation: In the data collection stage, behavior annotation method is adopted in field research to record visitors’ behaviors and interactions with their surroundings in an unobtrusive manner. Activity types, dwell durations, and spatial interactions are recorded without interfering with participants’ behaviors, and they can be used as a reference and data support for pedestrian simulation and visual perception experiment investigations, as well as compared to social media data to screen for DP nodes.

**FIGURE 3:**
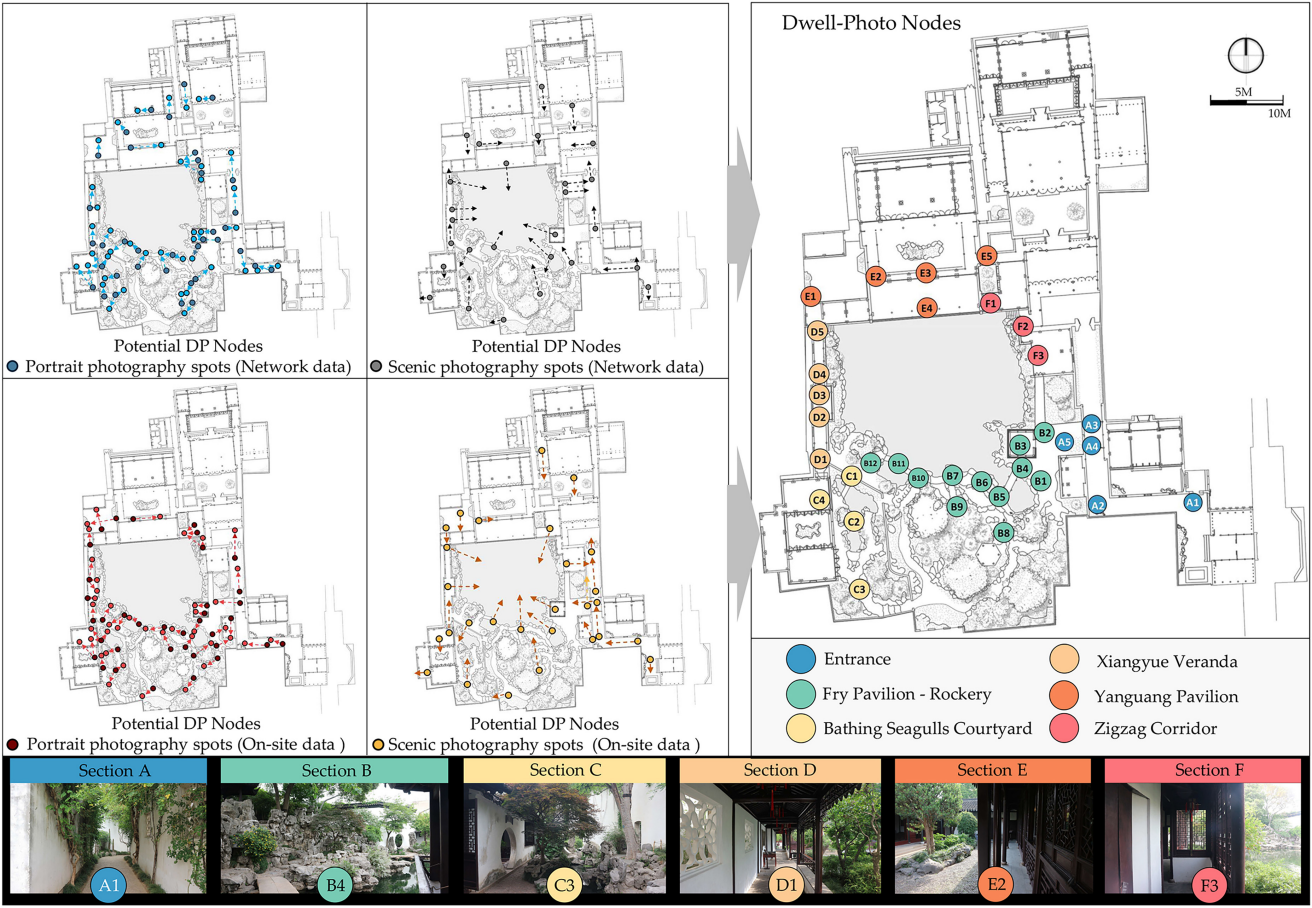
DP nodes.

Through a systematic analysis of visitors’ photographic content, 112 spatial nodes were identified, including 72 primarily portrait-oriented and 40 primarily landscape-oriented. The nodes were ordered in descending order of photography frequency, and nodes that overlapped geographically were combined. After removing 26 low-frequency nodes, a final set of 34 representative DP nodes was chosen for future investigation. As illustrate in Figure 3, Yipu was then separated into six major sections based on geographical continuity and functional zoning, and the 34 DP nodes were assigned accordingly.

#### 4.2.2 Data collection

The social media data collection began in April 2025 and was finished successfully in May 2025. It should be noted, data quality varies significantly between platforms. While Weibo content is fragmented and frequently lacks clear spatial references, platforms such as TikTok/Douyin and Bilibili are primarily focused on entertainment and performative content, making it difficult to effectively isolate and identify data specific to DP behaviors in classical gardens. Image-text uploads to RED provide a clear link between visitor perception and garden spatial organization. These postings employ easily comprehensible graphics to convey a variety of spatial and sensory information. As a result, RED serves as the study’s primary data source. The crawling method then concentrated on the primary keywords “Yipu,” “photography,” and “check-in” in order to selectively filter and gather pertinent user postings that were posted on the RED platform between January 2024 and May 2025. In the specific operation process, synonym supplementation and expansion methods are used to optimize keyword combinations, covering English synonyms such as “photo” and “hit up” and expanding the possible combinations of Chinese and English keywords to avoid data omissions caused by language differences. And select the “Remove Ads” option in the filtering process to avoid interference from advertising promotion information. Data gathering was carried out using Python crawlers to access RED’s open APIs, complemented by manual searches. A total of 1,247 initial data entries were collected. Subsequently, the collected data was subjected to quality screening and inspection.

① Identify and remove duplicate notes: Compare and filter the primary text and first image of the notes, examine notes that appear to be duplicates, and eliminate 189 notes.

② Geographic marker verification: Examine the geographic location markers and only maintain notes with accurate positioning of “Yipu” or specifically mentioning “in Yipu” in the text, removing 242 entries that merely mention the name but do not actually visit.

③ Integrity verification: The notes must include at least one on-site photo and a 10-word text description, as well as complete records of interaction data such as likes, favorites, and comments.

Following quality control criteria and the integration of multidimensional data, including images and accompanying text, and engagement metrics (like likes and saves), 346 sample notes with complete image-text and remark records were retained. This carefully chosen corpus serves as the empirical basis for the identification of DP nodes.

Based on social media data, text semantic analysis can identify key spatial environmental factors closely associated with tourists’ photo-check-in behavior, offering methodological guidance for data collection in behavioral annotation. The word frequency filtering method was used to extract fundamental variables: Light Environment, Pathway Scale, Lateral Enclosure Ratio, Overhead Enclosure Ratio, and Activity Density. These five elements represent sensory stimulation, spatial perception, ecological atmosphere, visual confinement, and behavioral interaction of tourists in garden area, providing a complete chain from physical environment to humanistic experience. The behavioral annotation and on-site data collection were carried out between April 1st and May 31st, 2025. The team collected data on the five elements and conduct targeted behavior annotation in conjunction with observations of tourist behaviors. To maximize sensory relevance (May is the peak growth period for garden flora) and thermal comfort (18–25 °C), as well as to avoid interference from extreme weather and important holidays, the May Day holiday was omitted, while the Dragon Boat Festival period was kept. The sampling schedule included weekdays, weekends, and public holidays, which allowed for the determination of average footfall for simulation studies.

Subsequently, the research team conducted standardized photography at each DP node to ensure the reliability and consistency of the visual perception experiment. In order to ensure lighting homogeneity and lessen the effect of shadow variation on the visual perception experiment, all photography shooting was completed between May and June 2025 with steady illumination. One representative image from each DP node was selected to serve as the primary visual stimulus for the perception experiment. The images were taken with a Canon EOS M10 Mirrorless Camera equipped with a 16-mm lens. Garden features including spatial layering, water reflections, and plant light-shadow interplay were prominently portrayed, and all photographs were standardized in aspect ratio, resolution, composition scale, and depth of field. These procedures made sure that the stimuli generated had good comparability for additional experimental investigation, balanced illumination, and significant instructive content.

### 4.3 Pedestrian simulation

**FIGURE 4:**
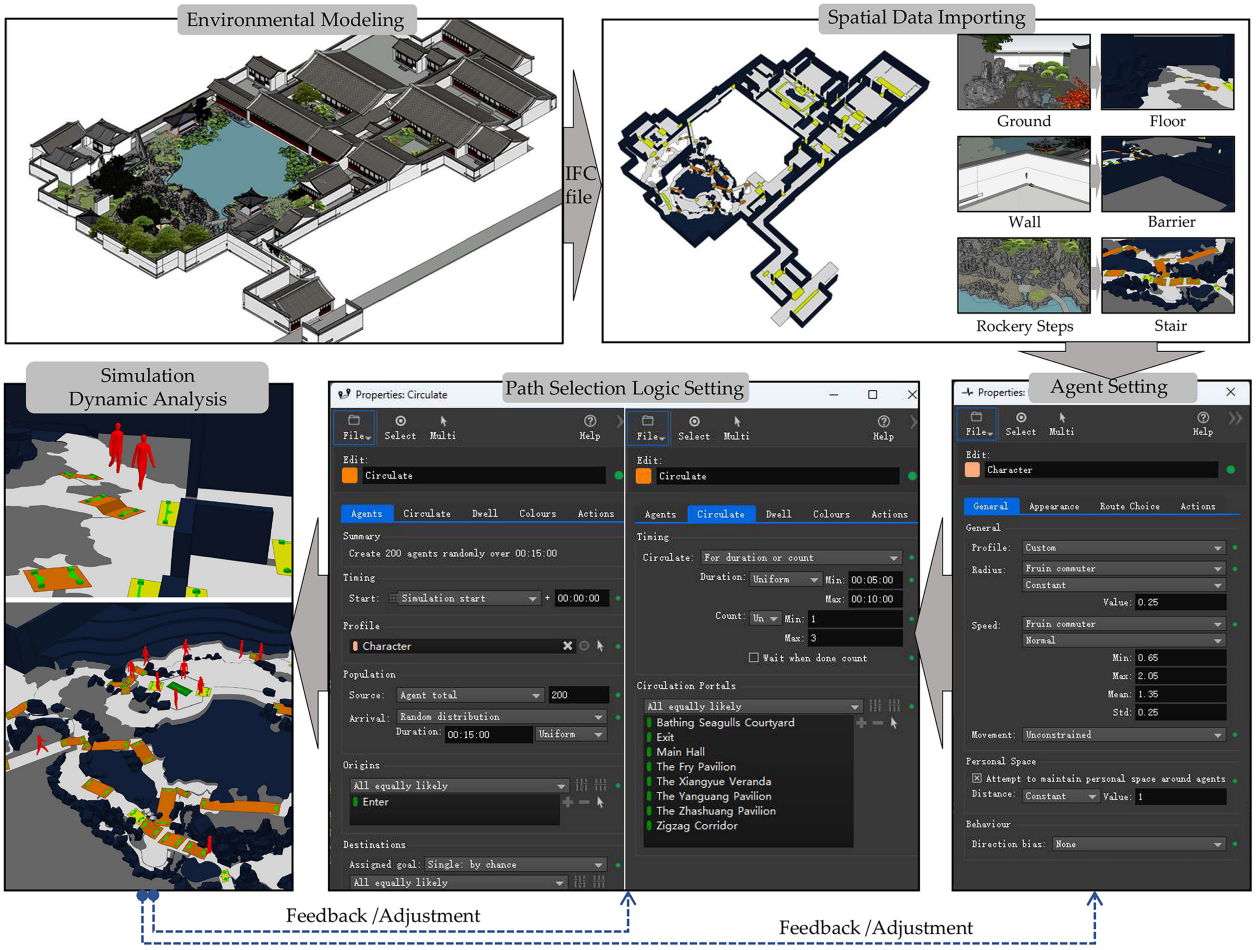
Pedestrian simulation.

Pedestrian simulation employs MassMotion software to accurately replicate tourist movement trajectories in the classical garden (Figure 4). This technique can expose characteristics of pedestrian flow, dynamically assess high-density gathering points or important traffic hubs, and provide supplementary data for the study.

#### 4.3.1 Modeling

A basic SketchUp 2023 model of Yipu was created in order to accurately mimic complex architectural and landscape elements, such as pavilions and rock formations (SketchUp (China), 2025). Key components, including ground surfaces and rockeries, were grouped (block-labeled) to facilitate classification and subsequent data administration. The model was exported as an IFC (Industry Foundation Classes) file to enable cross-platform interoperability (Arup, 2025).

#### 4.3.2 Spatial data importing

For semantic translation and refinement, the IFC model was loaded into MassMotion. To isolate the impact of spatial arrangement on behavior, only components directly associated with spatial organization were maintained during this process. The semantic mappings were defined as follows: ground and horizontal surfaces were converted to *Floor*; rockery steps were converted to *Stair*; walls were converted to *Barrier.* For walls containing windows, each section was uniformly defined to a 750-mm-high *Obstacle*, balancing visual continuity with functional separation. Subsequently, floor plates were subdivided according to the existing wall geometry, and spatial connectivity was established using the *Link function*. Entrances and exits were defined as *Portals*, and agent generation was constrained to these portal planes to ensure the accuracy and spatial validity of the simulation outputs (Mashhadawi, 2016; Oasys Software, 2025).

#### 4.3.3 Agent setting

Different virtual pedestrian types (e.g., adults, children, and the elderly) are created based on study data, and fundamental behavioral aspects like walking speed, range of view, and reaction time are built up to represent variations in the actual population. Based on on-site data, a total of 200 agents were used to estimate the pedestrian volume during the representative peak times of vacation mornings (09:00–11:00) and afternoons (15:00–17:00). Individual walking speeds in the Profile settings were drawn from a Normal (Gaussian) Distribution to better portray varied movement rhythms and replicate the inherent variety of tourist circulation patterns (Oasys Software, 2025; Wang et al., 2025).

#### 4.3.4 Path selection logic setting

Pedestrian entrances and exits (Origin) and target locations (Destination) were defined in the 3D model, and traffic statistics (such as pedestrian flow per minute) were configured to enable for dynamic modification over time. To imitate exploratory, non-goal-oriented visits, the pedestrian flow was set to *Circulate*, and each agent’s default behavior *(at birth)* was assigned the task *Seek Entrance/Exit* under the Actions. The *Random-Equal Probability* destination-selection option was set up to reflect the informal and experiential nature of visitor mobility inside the garden. Major picturesque locations and DP nodes were added to the *Entrance/Exit* list as possible movement targets in order to improve simulation accuracy. On the other hand, in order to prevent bias and preserve behavioral representativeness, resident agents associated with Yanguang Pavilion which display set, purpose-driven routines were not included in the simulation (Mashhadawi, 2016).

#### 4.3.5 Simulation and dynamic analysis

Pedestrian simulation calculations are carried out within the study area, and the relevant findings are presented. After analysis and screening, five essential variables with significant relevance to the study’s basic objectives were identified: Individual Count (IC), Maximum Density (MD), Individual Trajectory (IT), Visible Time (VT), and Visual Count (VC). These metrics are utilized to describe both the spatial distribution of visitors and the level of visual engagement, allowing for a thorough investigation of movement patterns and perceptual behaviors in the garden setting.

① IC (Individual Count) reflects the number of pedestrians present at various time intervals or spatial zones and is used to show the intensity of area utilization.

② MD (Maximum Density) demonstrates the concentration of individuals in a certain area at a given time period determines the likelihood of congestion and extended dwelling.

③ IT (Individual Trajectory) displays each pedestrian’s movement, which is used to describe patterns of route selection and the underlying structure of circulation flows.

④ VT (Visible Time) shows the length of time individuals stay in a certain area or field of view, which is an expression of how strong and persistent their visual engagement is.

⑤ VC (Visual Count) shows the number of fixations on a certain element or area, indicating the visual salience and significance in the scene.

### 4.4 Visual perception experiment

#### 4.4.1 Preparation

**FIGURE 5:**
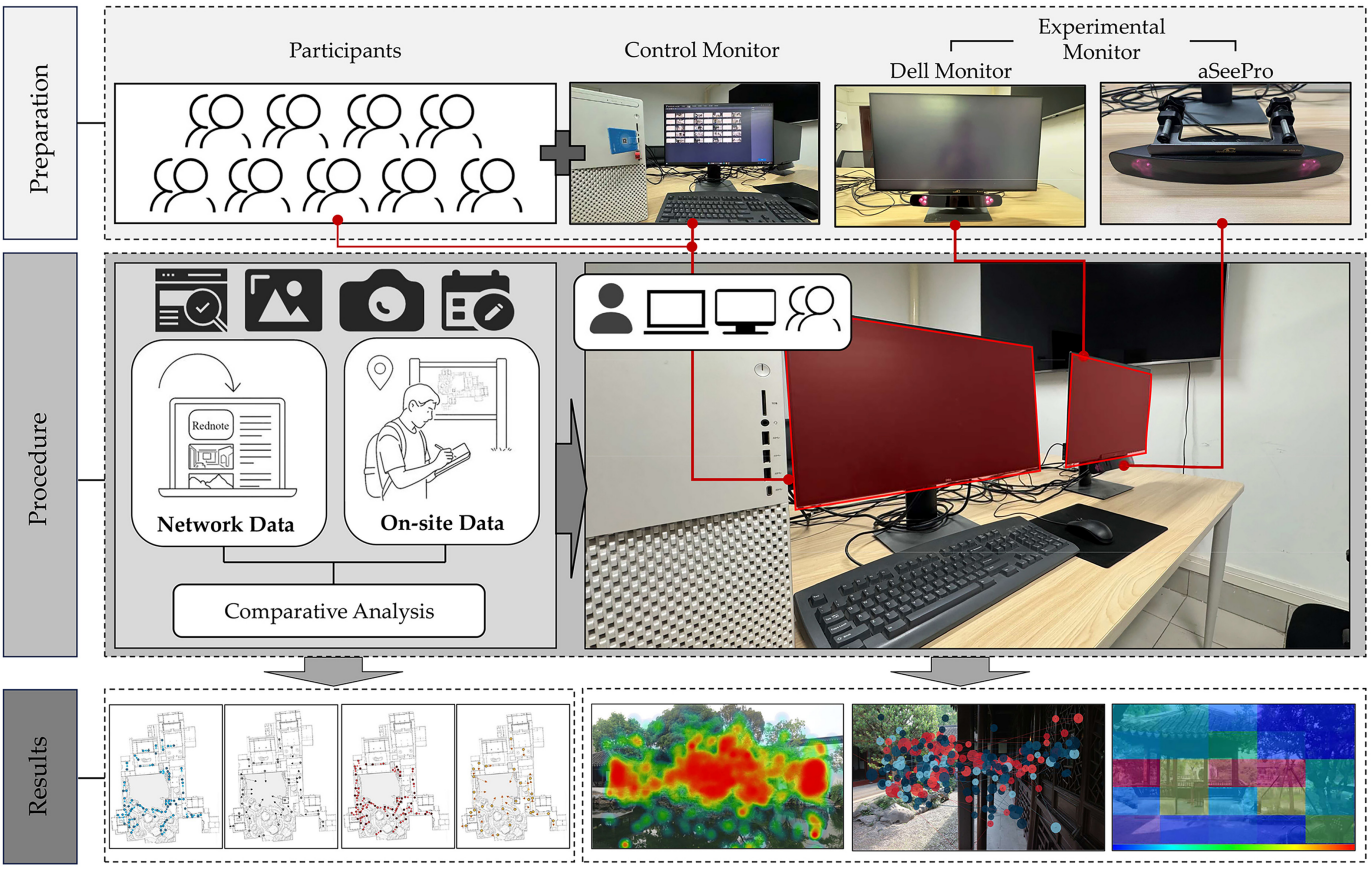
Visual perception experiment design.

The procedure of the visual experiment is illustrated in Figure 5. The experiment was carried out using a computer system connected to an external eye-tracking device (7invensun, 2025). The laboratory setup included a primary workstation connected to two Dell monitors: one for experiment control and real-time monitoring, and the other for participant usage, which was equipped with the aSeePro eye-tracking equipment for gaze data collecting. The device worked at a sampling rate of 100 Hz with an effective working distance of 50–80 cm. The external eye tracker has a head-movement range of 30 cm (W) × 25 cm (H) × 65 cm (D) and includes two EyeSensor modules for synchronized data collecting. The key components were a high-speed infrared imaging system, data transmission cables, and a power supply unit.

Photographs were processed correspondingly in Adobe Photoshop (2,025 release) to achieve a 16:9 aspect ratio in landscape orientation, a resolution of 2,880 × 1,616 pixels, and an image precision of 180 dpi (Adobe, 2025). To reduce potential bias during the experiment, all visible human figures were masked / anonymized without changing the scene content or diminishing visual information. During the perception experiment, the finalized images were exhibited as visual stimuli on a Dell monitor with a resolution of 1,920 × 1,080 pixel.

Operational viability, experimental reliability, and sample representativeness were taken into account while determining the total number of participants. Relevant literature shows that whereas psychology research usually considers samples larger than 30 to be statistically credible, landscape perception assessments typically involve 20–50 people (Zeng et al., 2024). As a result, the visual perception experiment involved 39 individuals. With an approximately 1:1 gender ratio, 10 were between the ages of 20 and 22, and 29 were between the ages of 24 and 30. In order to obtain accurate and dependable gaze data, participants were informed not to wear mascara or cosmetic contact lenses during the experiment, as well as to keep proper rest and a steady effect.

#### 4.4.2 Experiment and data

The visual perception experiment was divided into six sessions, with each lasting approximately 12 min and included 6–7 participants (Hoogerbrugge et al., 2025; Zhou et al., 2025). Images were shown sequentially, each for a predetermined duration (9 ≤ *t* ≤ 20). A two-second interval (blank screen) was placed between each succeeding image. Eight variables were selected for analysis:

① FH Map (Fixation Heat Map) depicts the density and spatial distribution of participants’ gaze concentrations. Higher heat values suggest greater visual appeal and spatial significance within the overall landscape composition, assisting in the identification of key focal points and explaining how spatial composition drives visual attention.

② FT Map (Fixation Trajectory Map) records the chronological sequence and spatial path of gaze shifts. Variations in the continuity or abruptness of trajectory lines reveal the organizational logic of visual flow.

③ TFD (Total Fixation Duration) records the total amount of time participants spend fixating. The degree to which informational richness and compositional complexity influence overall perception is reflected in longer TFD, which are thought to indicate stronger attentional involvement and higher cognitive-processing effort.

④ FFD (First Fixation Duration) measures the length of the initial fixation following onset. Shorter duration suggests rapid detection of salient features and efficient early-stage orientation, whereas longer duration imply greater effort to parse the initial focal elements.

⑤ FC (Fixation Count) shows the number of distinct fixations for each stimulus. Higher counts are regarded as reflecting more frequent attentional shifts and increased information retrieval activity, revealing greater image richness and more motivation for visual investigation.

⑥ FTD (Fixation Time Density) measures fixation time for each stimulus, standardized by display duration. Higher values indicate sustained attention independent of exposure length, supporting duration-independent comparisons of attentional intensity across scenes.


FTD=TFD/t

⑦NFF (Normalized First Fixation) indicates the ratio of first fixation time to total fixation time at the single image level. Higher values indicate a greater weighting of initial recognition within overall attention, highlighting elements that dominate early perceptual processing.


NFF=FFD/t

⑧FCR (Fixation Count Rate) records fixations per unit time for each presentation of images. Higher rates indicate faster-paced attentional shifting and accelerated information acquisition, providing a standardized basis for comparing viewing rhythm and cognitive throughput across scenes.


FCR=FC/t

## 5 Results

### 5.1 Features of DP nodes

**FIGURE 6:**
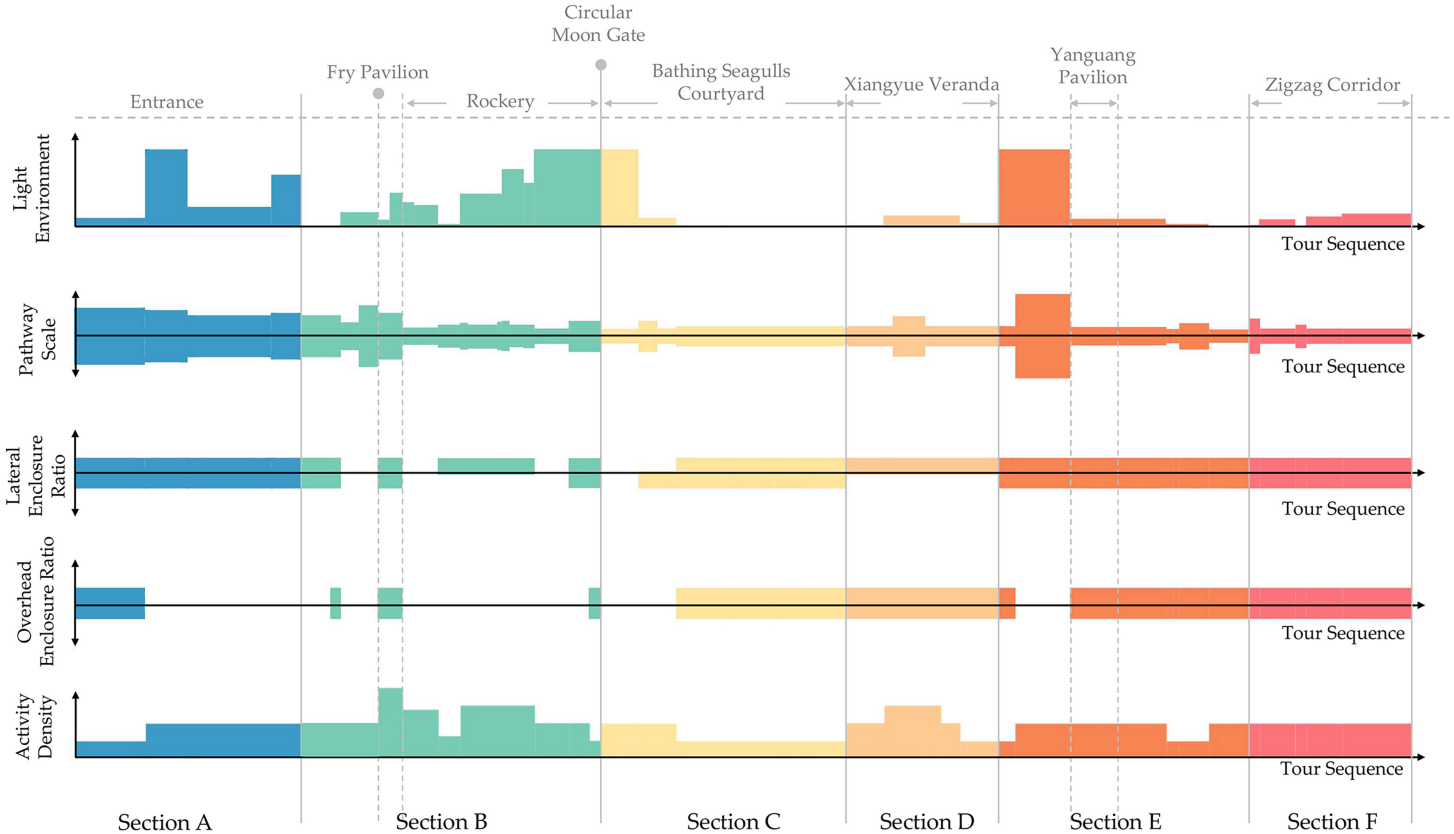
Features of DP node.

As illustrate in Figure 6, the features of DP nodes were primarily gathered through on-site observation, where each behavioral event’s duration, frequency, spatial trajectories, and ambient factors (such as illumination and facility layout) were meticulously recorded. These empirical records demonstrate the dynamic interactions between DP behavior and the constitutive spatial elements of the garden. As the research paradigm evolves from experience-based judgment to data-driven analysis, the generated datasets can improve study reliability by cross-validating with social media data, while also serving as an empirical foundation for future experimental analysis.

① Light environment

Yipu’s light environment showed a clear spatial rhythm and a contrast ratio of roughly 1:643, with measured illuminance ranging from 202 lx to over 130,000 lx. It is possible to figure out that regions with a high frequency of light environment changes display dense DP behavior by comparing the light environment data with DP behavior density. The alternating zones of intense lighting and overhead shadowing in section D (Xiangyue Veranda) and section E (Yanguang Pavilion) enhanced the feeling of depth and spatial limits, often resulting in stare fixations and portrait photography. Section C (Bathing Seagulls Courtyard), on the other hand, had more diffuse reflection and consistent illumination, which led to shorter dwell periods and fewer photographic acts. Although illuminance levels in section B were generally lower than in sections C and D, water-surface reflections and half-lit, semi-shaded pavilion interiors provided indirect illumination, which improved contour recognition and increased the likelihood of framing and photographing within narrow corridors.

② Pathway scale

Pathway dimension variation is crucial for modulating the rhythm of experience. Pathways in classical gardens are typically narrow, and there is a positive association between “scale compression” and “slowing of the visitor’s pace.” Narrow linear environments are found to focus sightlines and greatly restrict walking speed, resulting in DP behavior. Section B, the narrowest section with an average width of 0.8 meter, had the densest DP behavior and the greatest peak crowd density. Sections A and C are wider and allow for better circulation, but have weaker dwelling tendencies, characterized by fast visual scanning and brief stops.

③ Lateral enclosure ratio

There is a negative correlation between the frequency of DP behavior and the quantity of obstacles on both sides of the road. Sections D and E have the highest lateral enclosure ratio, owing to their semi-enclosed spatial linkages formed by continuous wall surfaces and extensive vegetation. This arrangement creates a strong sense of spatial enclosure and directional guidance, which increases the chance of gaze fixation and localized clustering. Section A, on the other hand, has the least lateral restriction, with open spatial boundaries and vast sight fields, allowing visitors to move through faster.

④ Overhead enclosure ratio

The degree of Overhead Enclosure Ratio has a substantial negative correlation with sky openness. In contrast to urban settings, the Activity Density and the frequency of stopping and starting are positively correlated with the Overhead Enclosure Ratio in garden areas. In sections D and E, canopy and roof structures provide near-continuous overhead coverage, producing pronounced light-shadow variations that induce visual compression and rhythmic fluctuation, thereby increasing fixation likelihood and enhancing psychological immersion. By contrast, sections C and F exhibit higher sky openness and broader visual fields but lack distinct visual anchors, resulting in reduced dwell durations and lower fixation densities.

⑤ Activity density

**FIGURE 7:**
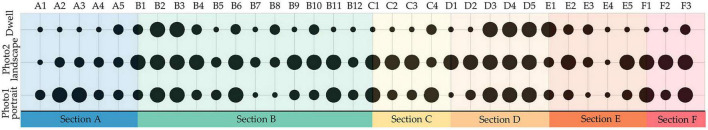
Activity density of each DP node.

As show in Figure 7, there is a coupled pattern of “high-density pedestrian flow” and “narrow-scale space.” Sections D and E contained more DP spots, with portrait photography accounting for more than 60%. During peak hours, Section B was the busiest. The shade and foreground of the Fry Pavilion, as well as the straight direction of the revetmentbridge axis, encourage visual focus and DP activities. Section C demonstrated sluggish mobility and frequent photographing in an open, uniformly illuminated space with mild restriction. Section D’s overhanging shade structure and light and shadow pattern attracted visitors to pose for photographs. Section A Section F functioned largely as a transitional and dispersal space.

### 5.2 Pedestrian simulation

**FIGURE 8:**
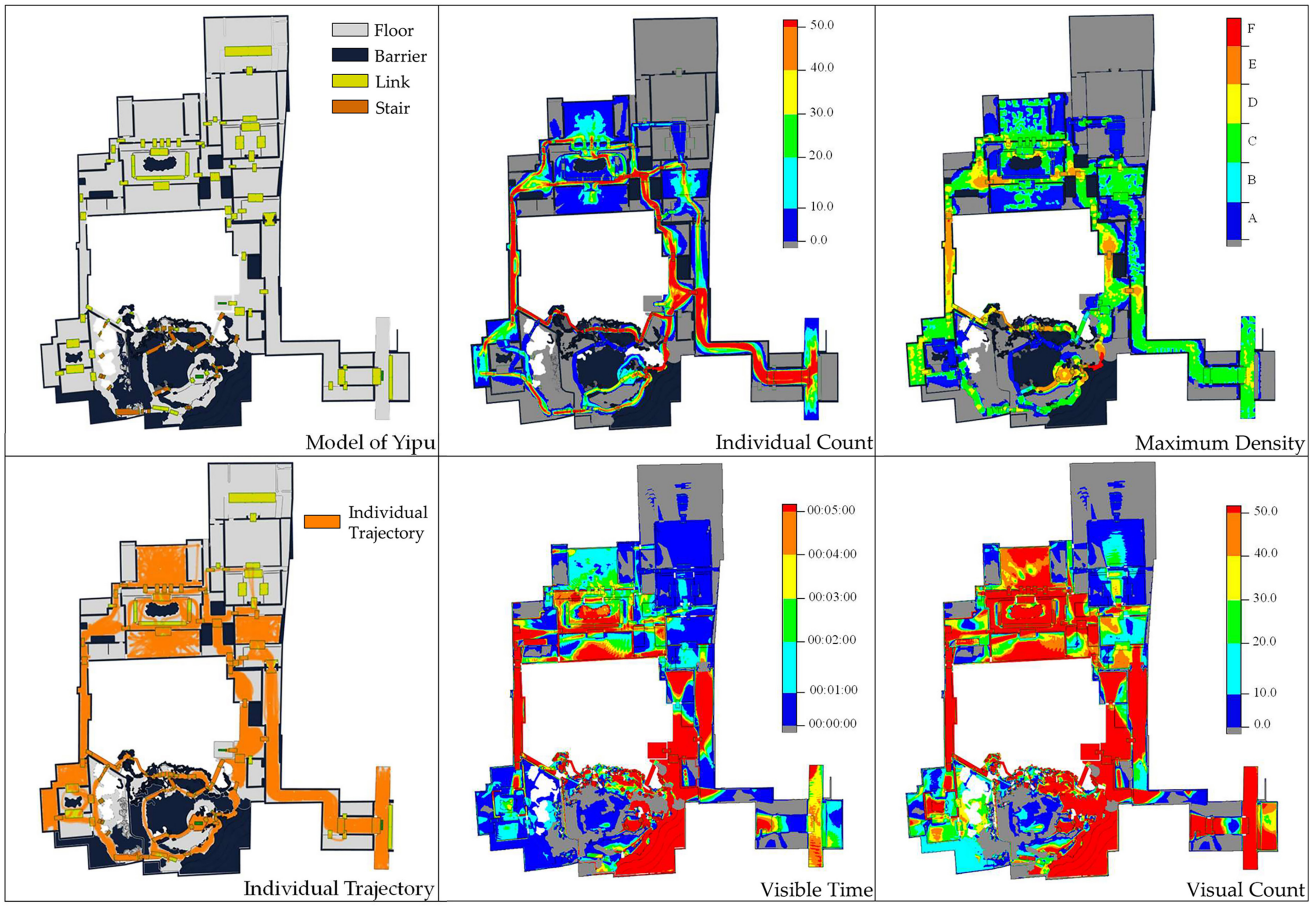
Pedestrian simulation.

The pedestrian simulation revealed patterns of gathering and dwelling in the garden area, indicating differences between spatial sections (Figure 8). These findings provide important references for identifying and characterizing DP nodes. The simulation aligns with the typical spatial order sequence of “Compression, Release, Turn (or Twist), and Diffusion” in classical gardens, displaying a dynamic pattern of concentration near the entrance, dispersion within central courtyards, and reconvergence along terminal corridors, capturing the rhythmic circulation logic of classical garden design. It should be noted that the black area in Figure 7 represents a barrier area that is inaccessible to visitors and has no significance on the calculation findings.

① IC (individual count)

Continuous high-value zones (IC ≥ 50) were concentrated along the southern water loop, extending to Xiangyue Veranda -Yanguang Pavilion section, with local peaks near the entrance and Circular Moon Gate nodes. By contrast, the northern courtyard and the northwestern residential quarter within the architectural precinct exhibited generally low values (IC < 25). This spatial pattern corresponds to the garden’s “water-axis” touring sequence, indicating that the origins, key nodes, and termini of the main circulation route foster localized dwelling. These results align with the distribution of DP nodes derived from social-media mining and with on-site field observations.

② MD (maximum density)

The distribution of the high density showed a “point-belt” pattern, with prominent peaks at turning points in section D and along the 0.8-meter rockery walk, bridge entrance, and Circular Moon Gate in section B. The courtyard in Section C and the northern corridor, on the other hand, displayed relatively low MD. When space compression and visual emphasis come together to heighten habitation and gathering behavior, these high-density zones usually correspond with geometric constrictions and VC value, especially door openings and framed-view compositions.

③ IT (individual trajectory)

The distribution of IT reveals the structural logic of experiencing circulation in the garden. The plotted breadth of paths (representing frequency of occurrence) defines a primary spine from sections A-B-C-D-E, with a distinct bifurcation and narrowing at section F, where routes turn and scatter. This trajectory rhythm, defined by commencement, continuance, transition, culmination, and diffusion, is similar to the traditional touring pattern and overlaps with portrait and landscape-photography chains as reported in behavioral annotations. Spatial zones with dense IT have high IC value and medium-to-long dwell duration, indicating an alternating pattern of walking and pausing along the main circulation axis that reflects the rhythmic balance of movement and contemplation that is central to classical garden experience.

④ VT (visible time)

While lower VT value parts were mostly found at the entrance zone and along the northern transit corridors, high VT value areas were located around the southern waterfront terrace and the Xiangyue Veranda-Yanguang Pavilion axis. This distribution suggests that spaces with semi-enclosed overhead structures and waterfront foregrounds extend the duration of both “seeing” and “being seen.”

⑤ VC (visual count)

The highlighted areas of MD and VT showed an outstanding correlation with the higher VC value zones. The VC value of the bridge entrance and Circular Moon Gate in section B, the column-revetment interface in section D, and the waterside corridor in section E were all of high level (VC > 50). These areas commonly combined horizontal revetment lines with vertical column-door sequences, forming a stable horizontal-vertical compositional framework. Correspondingly, heat maps indicated that fixation hot spots were continuously distributed along these linear spatial interfaces, underscoring their role as key visual attractors in structuring gaze and framing behavior.

### 5.3 Visual perception experiment

**FIGURE 9:**
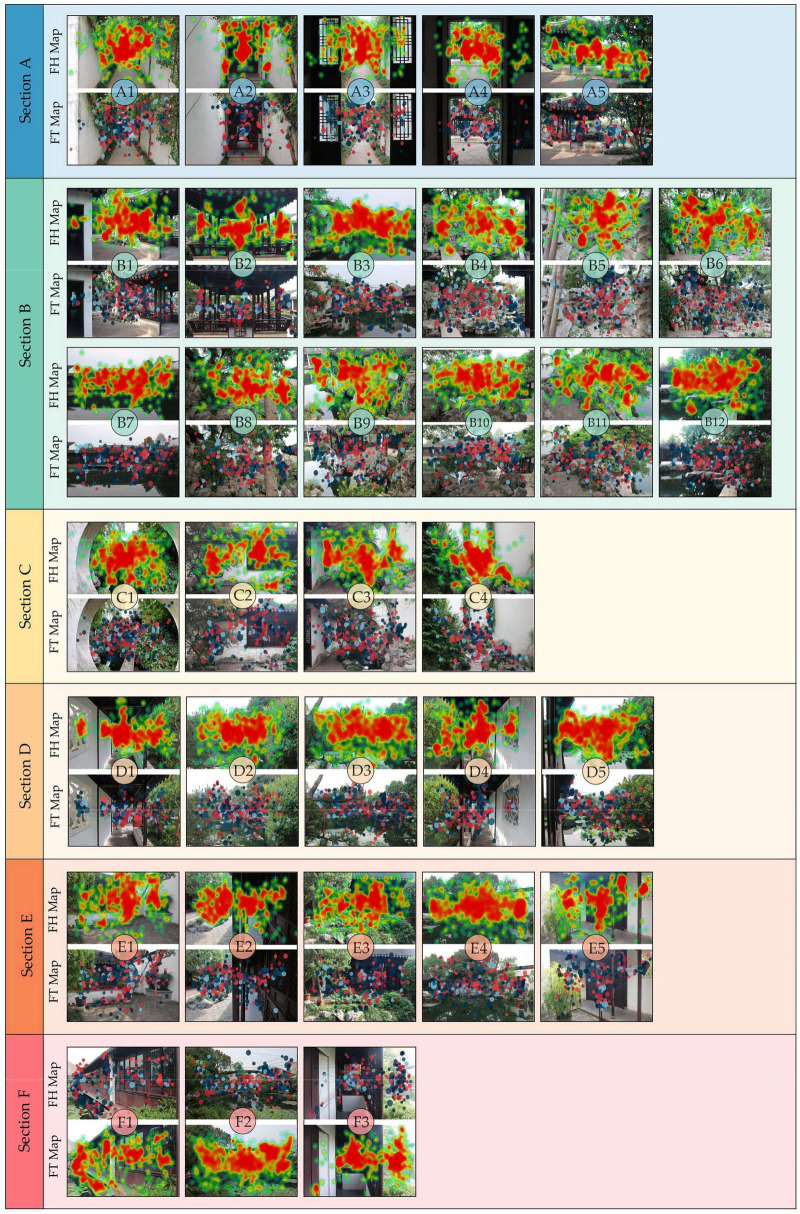
FH maps and FT maps of each DP node.

The visual perception experiment, based on eye-tracking, displays visitors’ patterns of visual concentration and cognitive rhythm throughout distinct spatial regions of Yipu (Figure 9 and Table 1).

**TABLE 1:** The visual perception of each DP node.

DP node	Playback duration	TFD	FFD	FC	FTD	NFF	FCR
A1	9	5.61	0.32	18.72	0.62	0.036	2.08
A2	9	5.92	0.42	16.76	0.66	0.047	1.86
A3	9	5.85	0.41	17.71	0.65	0.046	1.97
A4	10	6.74	0.35	19.93	0.61	0.035	1.99
A5	11	7.15	0.25	20.86	0.65	0.023	1.90
B1	11	6.61	0.32	22.71	0.60	0.029	2.06
B2	12	7.33	0.25	22.62	0.61	0.035	1.89
B3	20	11.92	0.29	39.54	0.60	0.023	1.98
B4	11	6.99	0.32	22.45	0.64	0.027	2.04
B5	10	6.08	0.29	21.75	0.61	0.023	2.18
B6	10	6.09	0.30	21.13	0.61	0.027	2.11
B7	10	5.99	0.27	21.17	0.60	0.026	2.12
B8	10	5.71	0.26	22.29	0.57	0.026	2.23
B9	11	6.61	0.30	24.41	0.60	0.027	2.22
B10	8	4.63	0.29	17.63	0.58	0.036	2.20
B11	10	5.97	0.27	22.58	0.60	0.027	2.26
B12	15	8.93	0.24	31.29	0.60	0.016	2.09
C1	13	7.84	0.28	27.13	0.60	0.022	2.09
C2	10	6.01	0.27	21.83	0.60	0.027	2.18
C3	12	7.01	0.26	25.92	0.58	0.022	2.16
C4	10	6.15	0.34	19.75	0.61	0.034	1.98
D1	10	6.57	0.26	21.46	0.66	0.019	2.14
D2	15	9.84	0.29	31.75	0.66	0.019	2.12
D3	20	11.62	0.23	42.00	0.58	0.012	2.10
D4	11	7.01	0.29	23.38	0.64	0.026	2.13
D5	20	11.29	0.26	38.79	0.56	0.013	1.94
E1	10	5.84	0.35	21.67	0.58	0.035	2.17
E2	10	6.09	0.32	20.75	0.61	0.032	2.08
E3	10	6.17	0.27	20.58	0.62	0.027	2.06
E4	20	11.92	0.29	40.08	0.56	0.015	2.00
E5	10	6.11	0.18	20.67	0.61	0.018	2.07
F1	9	5.51	0.39	19.21	0.61	0.043	2.13
F2	20	11.41	0.28	40.04	0.57	0.014	2.00
F3	10	6.34	0.37	20.17	0.63	0.037	2.02

Overall, the mean TFD varies from 3.6 to 4.4 s, and the mean FC from 5.8 to 6.8 per image, demonstrating typically persistent visual exploration and cognitive involvement across all spatial sections. Nodes in sections B and D near bridgeheads, Circular Moon Gate, and corridor turns (e.g., B3, B8, D3, D5) consistently exhibit higher TFD and FC values, accompanied by strongly overlapping hot spots in the FH Map. These patterns are interpreted as indicating that geometric narrowing combined with framed-views tends to produce dual clustering of gaze and pedestrian flow. By contrast, nodes in the inner courtyard of Section C and along selected residential interfaces in section E (e.g., C2, C4, E1, E2, E3) display lower FC values and function more as background-browsing scenes. In section F, node F2 exhibits a renewed increase in both FTD and FC, associated with spatial reopening and the emergence of new framing opportunities. The entrance area (section A) and the inner portion of the Sub Garden (section C) exhibited the strongest performance across multiple visual-attention indicators. These findings indicate that both locations have a strong visual attraction under experimental viewing conditions, which is consistent with the high integration and nodal values observed in the spatial-simulation model.

Analysis of FFD and its proportion of total fixation time highlights the critical role of morphological salience in generating visual attention. In section B (the waterside rockery area), the average FFD was only 0.21 s, yet its proportion of total fixation time reached 32%, the highest value in the entire garden. These results suggest that the strong morphological contrast and reflective qualities of the water-rock composition are rapidly detected and localized by the visual system, thereby triggering immediate focus and spontaneous photographic behavior.

TFD implies an intrinsic relationship that semi-enclosed spaces and environments with complex light-shadow variations tend to elicit more refined visual analysis and deeper cognitive processing. These patterns of prolonged fixation and increased fixation density correspond closely with the pedestrian simulation results, which indicate that variable illumination conditions exert a decelerating effect on pedestrian movement, thereby reinforcing the link between lighting rhythm, perceptual engagement, and behavioral response.

The comparative analysis of FH Maps and FT Maps indicates that framed-views and horizontal-vertical composition are particularly pronounced at nodes such as the Circular Moon Gate, Fry Pavilion, and Xiangyue Veranda. FFD along door-frame edges and eave lines is approximately 30% higher than the adjacent spatial average, while FT Maps exhibit a continuous sweep from the aperture toward the background. Taken together, these patterns support the interpretation that the framed-views blurs near-far relations, compresses perceived depth of field, and produces a two-dimensional pictorial field. By contrast, the alternation of light and shadow in Xiangyue Veranda is associated with periodic shifts in fixation points. Its FTD is approximately 14% higher than that of the entrance section, corresponding to an observed extension of fixation duration and supporting the hypothesis that lighting rhythm exerts a synchronous regulatory effect on the temporal rhythm of visual attention.

### 5.4 Comparative analysis

The spatial distribution of Yipu’s DP nodes is clearly unequal. Spatial scale, openness, degree of enclosure, and lighting conditions are strongly associated with visitor clustering and photographic attractions, which are concentrated in sections B, C, and D, according to the comparison of pedestrian simulation and visual perception experiment. Narrow cross-sections and shaded interfaces more easily cause momentary dwelling activities, higher illuminance and wider openness generally make photography and framing easier. For instance, while section F serves as a rhythm-damping and dispersal phase where both attentional concentration and photographic intensity significantly decrease, section A primarily serves as a rapid-transit zone.

The pedestrian simulation data shows a negative correlation between pathway width and crowd density: section A, which is approximately 2.9 meters wide, mainly facilitates rapid movement, while section B, which is only 0.8 meters wide, reaches a MD of 1.6 with IC and VT approximately 30% higher than average. These variations imply that the likelihood of congregation is increased by nodal overlap and geometric constriction. Section C (Bathing Seagulls Courtyard) combines moderate density (around 1.3) with high VC (VC > 48), whereas Section E maintains a lower density of 0.8 yet still exhibits concentrated visual attention, suggesting that semi-private spatial conditions may be more effective than simple crowding in focusing perception. In section D, increasing overhead coverage and strong light-shadow contrast highlight a “light-shadow rhythm and perceptual deceleration” effect. Additionally, in sections B, C, and D, the VC values are positively correlated with the salience of linear interfaces such as revetments, column arrays, and framed openings, whereas section F displays low density and dispersed fixation patterns, functioning as a spatial decompression zone. Overall, width, enclosure, light rhythm, and interface articulation jointly influence pedestrian experience via four interrelated channels including circulation, dwelling, movement speed, and framing, resulting in Yipu’s continual behavioral feedback loop of “Compression, Release, Turn (or Twist), and Diffusion.”

The visual perception experiment provides additional evidence for the previously indicated interrelationships. High spatial integration and wide visual fields in sections A, B, and C resulted in a “high-density and high-frequency” pattern that was characterized by frequent scanning movements and quick focusing. Conversely, sections D and E, which are distinguished by semi-enclosed buildings and alternating light-shadow conditions, were linked to high TFD value and a “low-speed and high-concentration” perceptual rhythm. There was a typical “visual decompression” effect in the section F, which is an area of deflection and diversion with scattered sites of focus and decreased TFD value. In summary, the spatial rhythm of “Compression, Release, Turn (or Twist), and Diffusion” in Yipu supports a dynamic coupling between visual perception experiment, behavioral movement, and spatial organization, reflecting the complex coherence between design intention and embodied experience in classical garden space, according to the strong correlation between pedestrian simulation results and FH Map distributions.

## 6 Discussion

In the sensory dynamics of classical garden spaces, the distribution of DP nodes is closely related to spatial scale, light-shadow rhythm, and interface morphology. Classical gardening techniques and place-making practices, such as rhythm management, light manipulation, and visual guidance, create a sensory environment that guides and encourages DP behavior. Pedestrian simulation reveals that the structural characteristics of spatial components directly influence their distribution by identifying potential spots where DP behavior is most likely to occur. The significance of framed-view design, horizontal-vertical composition, and light-shadow modulation for forming the overall spatial experience of a classical garden is confirmed by visual perception experiment.

### 6.1 Psychological mechanism of DP behavior triggered by spatial features and visual perception

#### 6.1.1 Attention capture: visual salience’s guiding function

According to environmental psychology’s Attentional Capture Theory, cognitive resources will be given priority to things in the environment that have a high visual salience. As defined by neuroscience, these spatial shifts activate the brain’s orbitofrontal cortex and amygdala, resulting in feelings of pleasure and curiosity.

DP Nodes in gardens with features like framed views, horizontal and vertical compositions, and light-shadow contrasts frequently become focus points in tourists’ images, according to research data. Eye-tracking research provides additional evidence that these nodes have the capacity to “capture attention.” In an effort to convert visual impact into enduring memories, travelers effectively “lock” these moments of focus through the lens when shooting pictures. It should be pointed out that this process is further strengthened by the spread of social media, which encourages travelers to pause and document this visually appealing picture (guiding DP behavior).

#### 6.1.2 Place attachment: spatial meaning projected emotionally

The notion of Place Attachment argues that people have different levels of emotional attachment to their surroundings, and that specific spatial nodes in gardens that have particular cultural connotations or personal recollections serve as carriers of emotional projection for visitors. The psychological basis of place attachment is this sentimental empathy evoked by location. From the standpoint of perceptual processing, visitors’ hippocampi are activated by cultural emblems in classical gardens, recalling cultural memories and feelings. When shooting pictures, tourists inadvertently incorporate their feelings onto the environment in an attempt to convey this emotional bond with the location. Place attachment in particular activities is demonstrated by this emotion-driven DP activities.

For example, the Yanguang Pavilion of Yipu was originally a daily gathering place for literati and neighbors to enjoy morning tea. This life-oriented historical memory causes modern visitors to unconsciously immerse themselves in a collective vision of “slow time” upon pausing. Visitors who take photos here not only capture the environment in front of them, but also connect emotionally with traditional culture through the lens.

#### 6.1.3 Group identity: behavior driven by social and cultural factors

Group identification influences visitors’ DP activities as well. Visitor photography activity reflects both personal perspective and collective identity. In gardens, visitors are frequently influenced by online information, peers, tour guides, and other visitors to select photo sites and tactics that are typical with the group. This group identity will encourage visitors to change their thoughts and conduct in order to comply to the group’s cultural norms and aesthetic standards. In the age of social media, visitors photo-taking habits are impacted by internet culture. They will replicate internet celebrities’ photo-taking techniques and select prominent spatial nodes on the internet to shoot images in order to obtain attention and likes from others.

### 6.2 Visual perception of spatial elements and its impact on dp behavior

#### 6.2.1 Perceptual functions of framed-views

The framed-view, achieved through doorways, lattice windows, corridor frames, and related interface elements, is a traditional compositional device that channels sight lines and reconstitutes visual scenes, guiding visitors to focus their gaze and pause briefly (Fung and Jackson, 1996; Girot and Imhof, 2017). Framed-view design (e.g., through circular apertures) is found to have the most significant effect on encouraging DP behavior by reinforces spatial stratification and rhythmic perception. Field investigations and social media data indicate that turning nodes along winding passageways and circular moon gates are among the most popular nodes for dwelling and photography. As shown in Table 2, While eye-tracking investigations reveal that door-frame edges and eave lines correspond to high-frequency fixation zones, with FTD value 37% higher than surrounding paths, pedestrian simulation results indicate that these locations have slower walking speeds and longer dwell duration. Framed views not only serve as physical constraints on visual composition, but they are also the key spatial stimulus for DP behavior in the traditional garden experience.

**TABLE 2:** The visual perception of framed-view landscape.

DP node	A2	A4	A5	C2	C4	F5
Schematic diagram	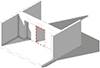	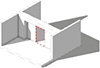	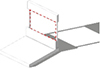	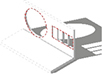	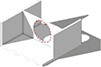	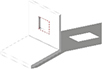
Framed-view landscape	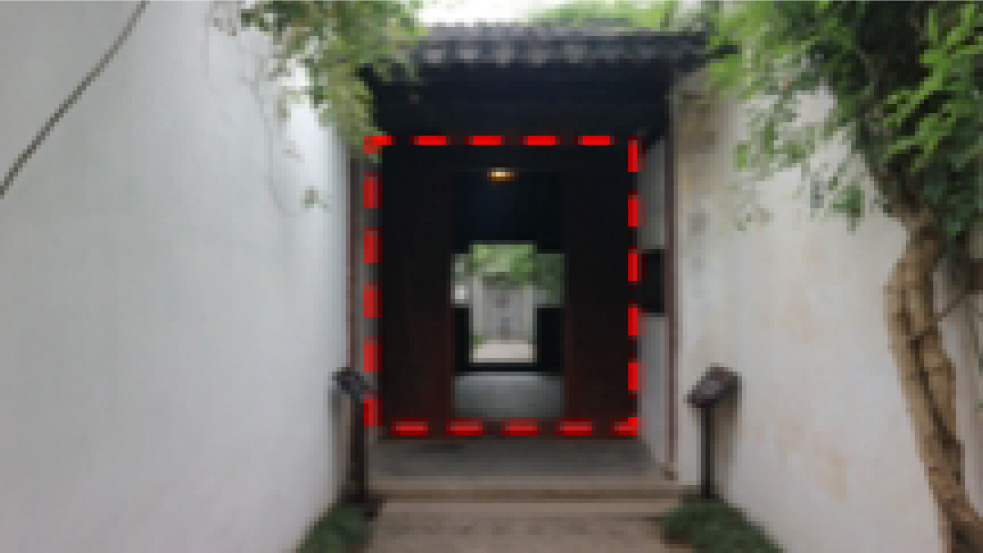	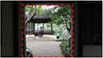	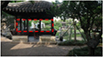	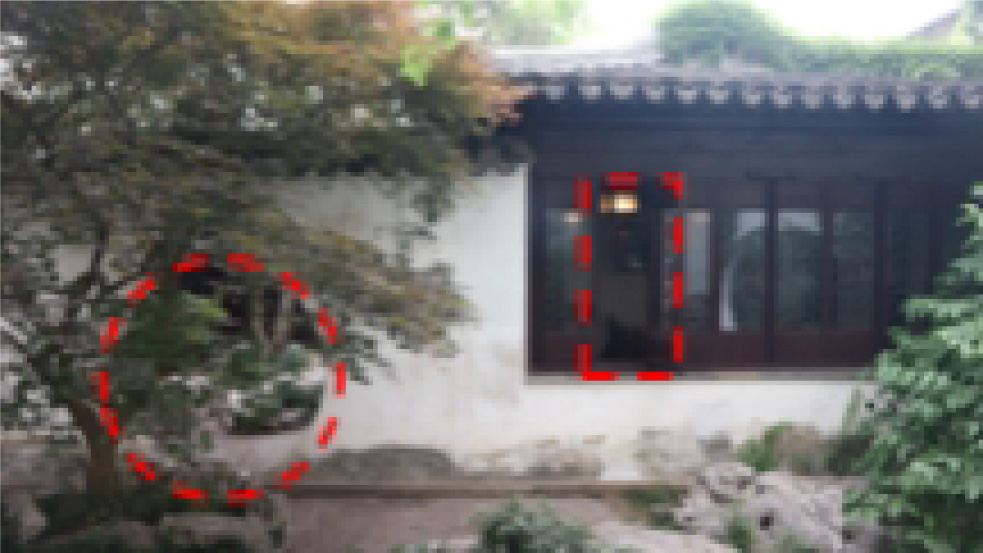	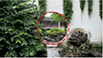	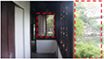
TH map	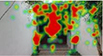	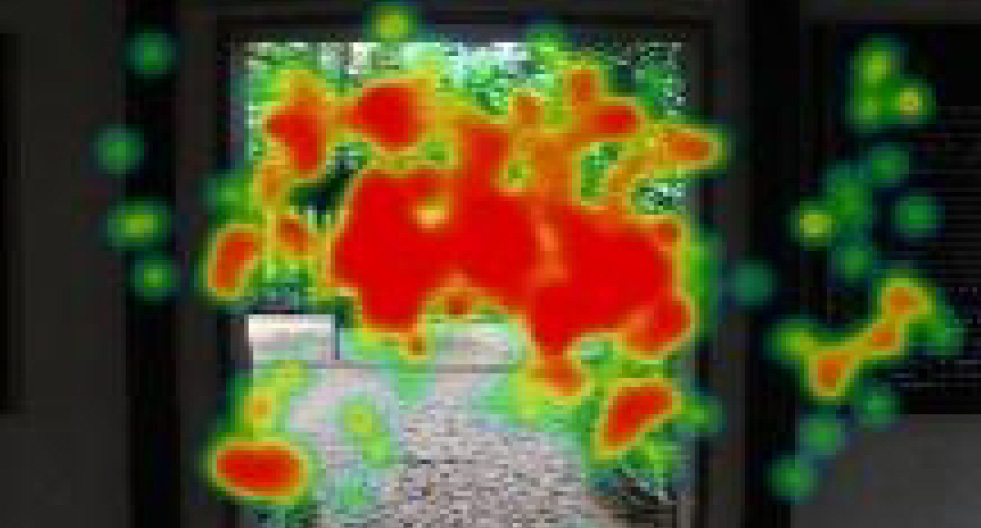	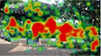	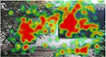	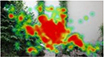	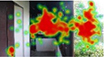

Additionally, there is a clear participative mechanism in the spatial experience of the framed-view compositions found in classical gardens. People’s visual engagement is enhanced by framed-view, which produces a “living painting” effect (Jullien, 2023; Li, 2023). By boosting on-site experience interaction and growing into a trans-spatial and temporal ritual of co-presence via social media, this mechanism turns classic gardens into dynamic “living visual fields.” This compositional approach constantly encourages visitors to “enter the picture,” which has a lasting impact on visual attention and touring behavior in the dynamic tension between roaming and viewing (Lash, 2023). Social media research indicates that over 30% of visitor-taken DP check-in photographs are framed-view compositions.

#### 6.2.2 Perceptual dynamics of horizontal-vertical composition

Landscape with complete horizontal or vertical compositions have a high visual appeal and are recognized as important spatial triggers for DP behavior in the classical garden experience. A delicate balance between horizontal and vertical spatial composition was sought, as articulated in the *Urban-Site Garden* chapter of *The Craft of Gardens*. In horizontal composition, linear features (e.g., bridges, waterfronts) provide visual breadth and lateral extension, while in vertical composition, elements (e.g., lattice or bamboo walls, tall trees) emphasize spatial stratification and vertical hierarchy (Corner and Hirsch, 2021; Kim et al., 2024). It should be pointed out that, owing to constraints of garden scale and design conventions, horizontally composed scenes are more prevalent than vertical ones and play a more substantial role in supporting and intensifying DP behavior within the overall experiential sequence of the classical garden.

A controlled foreground, a stretched midground, and a diffusive backdrop make up the fundamental rhythm of visual perception experiment and behavioral movement in Yipu’s spatial system. As illustrate in Table 3, the highlighted areas in the FH Maps were concentrated around bridge-revetment junctions, with the western and southeastern corners having the greatest FTD value (0.60) and a medium FC value (above 2). In comparison, the FTD value in the northern and eastern sectors is slightly lower (0.58), showing that more scanning motions across flatter surfaces are needed to achieve perceptual correction. When compared to the FH Map, the visual sequence of a centered foreground, wide midground, and permeable backdrop closely follows compositional tendencies and on-site photographic orientations identified in visitors’ images.

**TABLE 3:** The visual perception of horizontal/vertical composition.

DP node	DP check-in photo	FH map	FT map	Position	FTD	NFF	FCR
B3	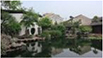	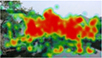	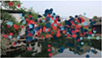	Western	0.60	0.023	1.98
B10	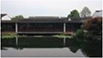	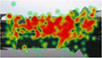	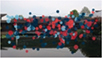	Northern	0.58	0.036	2.20
B15	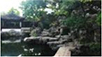	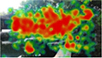	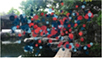	Southeast corner	0.60	0.016	2.09
D3	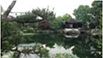	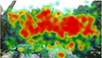	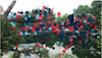	Eastern	0.58	0.012	2.10
E4	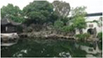	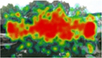	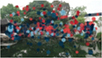	Southern	0.56	0.015	2.00
F2	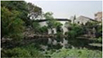	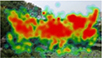	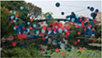	Southwest corner	0.57	0.014	2.00

By encouraging visual extension and association, the horizontal and vertical composition of classical gardens strengthens the landscape’s sense of hierarchy and creative notion, increasing its appeal to tourists. It not only improves visitors’ visual experiences but also lengthens their stays and piques their curiosity, all of which are essential for the experiential upgrading of contemporary tourism (Schänzel and Apollo, 2023). On the one hand, horizontal and vertical construction maps can provide distinct visual symbols and memory spots, becoming one of the primary motivators for visitors to “check in” (Wang et al., 2022). This may boost the attractiveness and recognition of picturesque areas through media transmission. On the other hand, the visual extension of horizontal and vertical composition can direct tourists’ dynamic viewing and stimulate a willingness to shift from “scenic spot check-in” to “immersive experience,” which proves crucial for the experience upgrading of the current tourism industry.

#### 6.2.3 Behavioral activation mechanisms of light-shadow rhythm

Within the spatial structure of classical gardens, the rhythm of light and shadow is a crucial mechanism for evoking DP behavior. Light and shadow are carefully coordinated to create a legible spatial language that runs throughout the construction, perception, and dissemination phases. Form, scale, surface boundaries, and material reflectivity are used to transform light into an implicit medium that organizes spatial rhythm and guides visitor behavior. In the media era, tourists can continuously analyze and maintain the perceptual dimensions and aesthetic values carried by light and shadow by recording photographs and sharing content. Thus, light and shadow serve as both environmental factors and behavioral triggers in the classical garden, facilitating the ongoing feedback loop between spatial layout and sensory perception.

The visual perception experiment provided more evidence for the validity of the “Light-shadow rhythm stimulates DP behavior” hypothesis. Both online and on-site photographic records confirm this pattern: compositions frequently use the brightness-darkness border as the primary visual axis, and high-frequency visitor images are focused at light-shadow transition nodes.

As described in *The Craft of Gardens*, “the scene is derived from the form itself,” site orientation, solar exposure, eave depth, and aperture location were precisely calibrated to generate a unique visual rhythm based on diurnal and seasonal light variations. Light-shadow rhythm in gardens can significantly increase the poetic and immersive experience of the landscape by shaping dynamic visual perception and emotional ambiance, making it a key design feature for improving tourism appeal and lengthening stay time., This is congruent with research on the “experience economy” and “sensory marketing” in tourism studies(Pukowiec-Kurda and Apollo, 2024). The memory anchor is deeper, the emotional connection is greater, and it is more likely to result in word-of-mouth communication and a desire to return (Apollo et al., 2025). This natural and humane fusion of “light narrative” not only enhances travelers’ sensory experiences, but also encourages the spontaneous diffusion of a vast volume of user-generated content, exhibiting tremendous value in cultural communication and commercial transformation. For the tourism industry, this low-cost and high-return design approach can achieve spatial experience upgrades through plant configuration, path optimization, and other methods, as well as combining natural and artificial light to construct a day-night full-time tourism model; when deeply integrated with other sensory stimuli such as hearing, unique cultural memory points can be created.

### 6.3 Limitations

This study takes an interdisciplinary approach, combining environmental psychology, architecture, and urban design. However, due to limitations in the research methodology and data dimensions, the current findings continue to focus on the surface impact mechanism of spatial features and their visual perception on the experience of visitors. Future research can overcome the limitations of a single perspective by delving deeper into the psychological core of tourist experience and systematically analyzing the deep path of spatial factors influencing visitors perception, decision-making, and behavior from psychological dimensions such as cognitive processing, emotional arousal, and motivation. This will provide more targeted scientific support for spatial optimization of tourist locations, scene creation, and the high-quality development of the tourism industry.

In terms of data collection, this study fully addresses the dissemination characteristics of geographical experience information in the digital age. However, algorithmic recommendation systems and user-group preferences may influence check-in data and “popularity” reasoning, thus leading to a divergence between apparent dissemination intensity and user experience. In order to address this limitation, this study employs the “dual-track verification” method, which entails completing a two-month field survey during the DP node identification phase. Cross-validating social media data involves in-depth interviews, focus groups, and participatory observation. To improve data quality, future research might involve a “dynamic weight adjustment model,” a time decay function for user behavior data, and a cross-platform data verification mechanism to validate findings with multi-source data.

The experimental framework effectively combines pedestrian simulation modeling with visual perception experiment analysis. It is nevertheless difficult to properly duplicate visitors’ actual states in different seasonal, climatic, festive, or crowd-density situations, even though variables were adjusted to match real-world circumstances. The pedestrian simulations and visual perception experiments were carried out in a controlled laboratory setting, which inevitably limited the capacity of the avatars to capture complex behavioral variables including mood changes and social interactions. Future studies might concentrate on cross-seasonal and multi-temporal mobile eye-tracking, wearable physiological sensors (including pupil diameter and heart rate), soundscape and micro-climate monitoring, and multi-platform UGC-GIS spatiotemporal alignment with longitudinal tracking.

The paradox between “dwell needs” and “congestion reality” in DP nodes was also found in this study. Although these nodes were initially intended to satisfy users’ needs for viewing and relaxation, their DP check-in behavior necessitates time and space, and when a large number of users flock in, congestion is frequently formed, which actually weakens the leisure atmosphere. In order to thoroughly examine the intrinsic connection between DP behavior, spatial carrying, and congestion creation, as well as investigate how to strike a balance between check-in demand and leisure experience, study scenarios and groups centered around this paradox can be broadened in the future.

## 7 Conclusion

The quality of visual experiences has a greater impact on tourists’ perceptions than ever before in the social media era. More visitors are encouraged to take part in these experiences through the sharing of visual information, such as travel photos. This effect is especially noticeable in classical gardens, and visitors’ DP behavior provides a crucial analysis lens for assessing compositional strategies in classical gardens. Comparative evaluations of pedestrian simulation and visual perception experiment indicate that DP behavior is guided by framed-view design, regulated by horizontal-vertical composition, and activated by light-shadow rhythm. Classical garden-making strategies such as entrance inflection, meandering corridors, and water permeability, in addition to framing, horizontal composition, and luminance contrast, serve as both directive and narrative devices; they shape users’ perceptions of spatial order and, in the context of digital dissemination, translate into a shareable visual language that connects perception and diffusion.

This study uses pedestrian simulation and a visual perception experiment based on behavioral annotation and social media data mining to propose a “space-behavior” cognitive paradigm that illuminates the spatial determinants and operational mechanisms behind the DP phenomena. The visual perception experiment connects spatial aspects to visual attention, whereas the pedestrian simulation connects spatial elements to DP behavior, exposing potential flow pressures and aggregation trends. When combined with fieldwork and social media data, the two procedures are mutually supportive and assist overcome the shortcomings of single-method research in demonstrating the interaction between spatial activity and perceptual cognition. It can provide scientific paradigms and decision-making references for the inheritance and innovation of classical garden spatial construction approaches, as well as urban streetscape aesthetic design. In response to the binary dilemma of protecting tourism destinations represented by traditional gardens, such as urban heritage and historic districts, which frequently falls into “static sealing” or “excessive commercialization,” spatial perception and tourist behavior data research can provide an accurate foundation for heritage revitalization.

This study introduces pedestrian simulation and visual perception experiments, establishes the connection between spatial elements and DP behavior, and gathers multi-modal data for systematic analysis based on conventional environmental behavior and scenery perception research. By identifying the DP nodes in Jiangnan classical gardens, the mechanism of DP behavior was examined, as well as the spatial components that encourage this activity. In addition to confirming the driving influence of environmental elements on behavioral patterns, this approach offers a methodical instrument for comprehending visitors’ behavior in the media era, which is particularly appropriate for the sophisticated study of cultural heritage locations including classical gardens.

The interdisciplinary integration path and quantitative model provided in the study can provide realistic scientific paradigms and decision-making references for the current cultural and tourism scenes, hence promoting the sustainable development of tourist destinations. The quantitative evaluation procedure connects spatial perception data (e.g., tourist gaze trajectory, duration of stay, and emotional feedback) to tourism planning and management plans. This provides a scientific foundation for landscape creation, facility layout, and streamline management, reducing resource waste due to subjective judgments and providing decision-making references for tourism planning.

## Data Availability

The original contributions presented in the study are included in the article/supplementary material, further inquiries can be directed to the corresponding author.
